# Effects of anti-inflammatory drugs on fever and neutrophilia induced by *Clostridium difficile* toxin B

**DOI:** 10.1155/S0962935196000245

**Published:** 1996-06

**Authors:** R. A. Cardoso, A. A. Melo Filho, M. C. C. Melo, D. M. Lyerly, T. D. Wilkins, A. A. M. Lima, R. A. Ribeiro, G. E. P. Souza

**Affiliations:** 1 Laboratory of Pharmacology Faculty of Pharmaceutical Sciences USP Campus-14040-903 Ribeirão Preto S.P. Brazil; 2 Department of Physiology and Pharmacology and Division of Infectious Diseases Clinical Research Unit Federal University of Ceará Fortaleza C.E. Brazil; 3 Department of Anaerobic Microbiology Virginia Polytechnic Institute and State University Blacksburg VA 24061 USA

## Abstract

This study investigated the ability of *Clostridium difficile* toxin B, isolated from the VPI 10463 strain, to induce fever and neutrophilia in rats. Intravenous injection of toxin B (0.005–0.5 μg/kg) evoked a dose-dependent increase in body temperature. The febrile response to 0.5 μg/kg of the toxin started in 2.5 h, peaked at 5 h, and subsided fully within 24 h. Toxin B also induced a dosedependent neutrophilia. Pretreatment with indomethacin (2 mg/kg, i.p.) did not affect the neutrophilia induced by toxin B, but significantly reduced the febrile response measured 4 to 8 h after toxin B injection. Dexamethasone (0.5 mg/ kg) also markedly diminished the febrile response induced by toxin B. These results show that *Clostridium difficile* toxin B induced a febrile response susceptible to inhibition by dexamethasone and indomethacin. Furthermore, they suggest that prostaglandins are not involved in the neutrophilia caused by this toxin.


Mediators of Inflammation, 5, 183–187 (1996)

### Effects of anti-inflammatory drugs on fever and neutrophilia induced by *Clostridium difficile* toxin B

R. A. Cardoso,[1] A. A. Melo Filho,[2] M. C. C. Melo,[1] D. M. Lyerly,[3] T. D. Wilkins,[3] A. A. M. Lima,[2] R. A. Ribeiro[2] and G. E. P. Souza[1,CA]

[1]Laboratory of Pharmacology, Faculty of Pharmaceutical Sciences, USP Campus-14040-903, Ribeirão Preto, S.P., Brazil; [2]Department of Physiology and Pharmacology and Division of Infectious Diseases, Clinical Research Unit, Federal University of Ceará, Fortaleza, C.E., Brazil; and [3]Department of Anaerobic Microbiology, Virginia Polytechnic Institute and State University, Blacksburg, VA 24061, USA

[CA]Corresponding Author

**This study investigated the ability of *Clostridium difficile* toxin B, isolated from the VPI 10463 strain, to induce fever and neutrophilia in rats. Intravenous injection of toxin B (0.005–0.5 μg/kg) evoked a dose-dependent increase in body temperature. The febrile response to 0.5 μg/kg of the toxin started in 2.5 h, peaked at 5 h, and subsided fully within 24 h. Toxin B also induced a dose-dependent neutrophilia. Pretreatment with indomethacin (2 mg/kg, i.p.) did not affect the neutrophilia induced by toxin B, but significantly reduced the febrile response measured 4 to 8 h after toxin B injection. Dexamethasone (0.5 mg/kg) also markedly diminished the febrile response induced by toxin B. These results show that *Clostridium difficile* toxin B induced a febrile response susceptible to inhibition by dexamethasone and indomethacin. Furthermore, they suggest that prostaglandins are not involved in the neutrophilia caused by this toxin.**

**Key words**: Anti-inflammatory drugs, *Clostridium difficile* toxin B, Dexamethasone, Fever, Indomethacin, Neutrophilia

### Introduction

Fever is an important component of the acute-phase response (APR), which is induced by exogenous pyrogens (bacterial products), and is related to their ability to stimulate cytokine production by host cells, e.g. macrophages, lymphocytes and endothelial cells. Many of these cytokines, including interleukin (IL)-1, IL-6, tumour necrosis factor-α (TNF-α), interferons (IFNs), IL-8 and macrophage inflammatory protein-1 (MIP-1), generally known as endogenous pyrogens (EPs), can induce fever when injected into experimental animals and humans.[1–5] It is likely that IL-1, IL-6, TNF-α and IFNs induce fever by stimulating synthesis of prostaglandins (PGs).[1] On the other hand, fevers induced by MIP-1 in rabbits and rats and IL-8 in rats are independent of prostaglandin synthesis.[2–5] Another important component of APR is neutrophilia, which is also dependent on synthesis and release of cytokines, including IL-1α and β, TNF-α and β, IL-6, IL-8, as well other inflammatory mediators such as C5a and PGs ($E_1$, $E_2$, $F_{2\alpha}$).[6–11]

*Clostridium difficile*, a Gram-positive anaerobic bacillus, is a common cause of diarrhoea associated with antibiotic therapy in elderly and debilitated hospitalized patients.[12,13] The clinical presentation of this infection is broad and ranges from an asymptomatic carriage to an acute abdomen and fulminant, life-threatening colitis.[14,15] Clinical symptoms of *C. difficile* colitis are profuse and debilitating diarrhoea, abdominal pain and distension. Furthermore, systemic features may also be present, such as polymorphonuclear leukocytosis, nausea, anorexia, malaise, dehydration and fever.[14] This last sign is clinically relevant in pseudomembranous colitis associated with the infection by *C. difficile*.[16–18] Also, it has been suggested that, in postoperative patients who develop diarrhoea, the presence of an unexplained fever or high white blood-cell count is an important clue to this.[19]

Although *C. difficile* is non-invasive, its toxins seem to permeate the mucosal barrier since they can induce an antibody response by the host.[20] To date, two *C. difficile* exotoxins have been described, toxin A (TXA) and toxin B (TXB), each encoded by distinct genes.[21–24] TXA causes fluid accumulation associated with mucosal damage in several animal models.[25] In contrast, TXB has no enterotoxic activity,[26–28] but it is 1 000-fold more potent as a cytotoxin than is TXA in tissue culture lines.[26,27] However, both toxins are potent proinflammatory agents that cause erythematous and haemorrhagic lesions in rabbit and guinea-pig skin.[26,27] In addition, both TXA and TXB induce release of cytokines from human monocytes, polymorphonuclear neutrophils and cultured epithelial cells.[29–31]

© 1996 Rapid Science Publishers

To our knowledge there is no experimental demonstration of the ability of toxins from *C. difficile* to induce fever and neutrophilia, although these signals occur with this infection. Hence, the aim of this study was to investigate the pyrogenic activity and the ability to induce neutrophilia of the *C. difficile* toxin B, in rats. In addition, we also investigated the effect of anti-inflammatory drugs in TXB-induced fever and neutrophilia.

### Materials and Methods

*Animals:* Wistar rats weighing 180–200 g were housed at 24 ± 1°C under a 12 h light/dark cycle (lights on at 06.00 h), and had free access to water and food.

*Temperature measurements:* Rectal temperature was measured by inserting a thermistor probe (Y.S.I. no. 402) 3 cm into the rectum. The animals were held manually in their cages during the temperature measurements. This procedure was performed at least twice on the day before the experiment to minimize stress-induced temperature changes secondary to handling. On the day of the experiment, the basal temperature of each animal was determined at least three times, at 30 min intervals, before any injection. Only animals with basal temperatures between 36.8 and 37.6°C were used. After the injection of the TXB or saline, the temperature of the rats was measured up to 6 or 8 h, at 30 min intervals. The experiments were conducted at the thermoneutral zone for rats (28 ± 1°C) between 08.00 and 19.00 h.[32]

*Purification of* C. difficile *toxin B:* TXB was purified to homogeneity from culture filtrate of *C. difficile* (VPI 10463 strain) as described previously.[33] Briefly, it was purified by ammonium sulphate precipitation, ion-exchange chromatography on DEAE–Sepharose CL-6B, and immunoaffinity chromatography. Homogeneity of TXB was assessed by crossed immunoelectrophoresis and polyacrylamide gel electrophoresis.[21]

*Experimental protocols:* TXB was administered intravenously (i.v.) via a tail vein at doses of 0.005, 0.05, 0.5 and 2.5 μg/kg in a volume of 0.2 ml. The effects of steroidal and non-steroidal anti-inflammatory drugs on temperature responses to i.v. injection of TXB were investigated. Indomethacin (2 mg/kg) was given intraperitoneally (i.p.) 30 min before the i.v. injection of TXB. Dexamethasone (0.5 mg/kg) was administered subcutaneously (s.c.) 1 h before TXB. Control animals were treated with the appropriate vehicle only. The injection of TXB was carried out between 09.00 and 10.00 h.

*Leucocyte counts:* Six h after TXB injection, animals were anaesthetized with pentobarbitone sodium (40 mg/kg, i.p.) and blood samples were taken from the abdominal aorta for determination of total and differential cell counts. These are reported as the number of neutrophils per ml of blood.

*Drugs:* Indomethacin was a gift from Merck, Sharp & Dohme, Brazil. Dexamethasone (Decadronal®) was purchased from Prodrome, Brazil.

*Statistical analysis:* For analysis of the rectal temperature data, the average baseline temperature before any injection was calculated for each animal, and all subsequent temperatures were expressed as changes from this average value. All values are reported as the mean ± S.E.M. The area under the curve was calculated for each animal and expressed in arbitrary units as an index for the magnitude of the febrile response over the period of measurements (fever index). The individual results were then combined to calculate the group mean ± S.E.M. and were analysed for statistical significance using one-way analysis of variance, followed by Tukey's test. The limit for the level of significance was set at $p < 0.05$.

### Results

Figure 1 shows that i.v. injection of *C. difficile* TXB (0.005, 0.05 and 0.5 μk/kg) induced a dose-dependent increase in body temperature. Similar to saline, the dose of 0.005 μg/kg promoted no change in body temperature, whereas the dose of 0.05 μg/kg induced a small but not significant increase (0.19 ± 0.10°C, 5 h). At the highest dose (0.5 μg/kg) the increase in body temperature started around 2.5 h, peaked at 5 h (0.97 ± 0.10°C) and returned to basal levels after 24 h (–0.13 ± 0.03°C). The fever index from this curve is represented in the right of Fig. 1 (saline, 0.29 ± 0.48; TXB, 0.005 μg/kg, 1.10 ± 0.64; 0.05 μg/kg 2.49 ± 0.41; and 0.5 μg/kg; 4.92 ± 0.81) and the correlation between the fever response and the doses of TXB was $r$ = 0.97. Furthermore, TXB also induced a dose-dependent neutrophilia 6 h after injection, which was statistically significant at doses of 0.05 and 0.5 μg/kg (saline, 1.3 ± 0.2; TXB, 0.005 μg/kg, 1.5 ± 0.4; 0.05 μg/kg, 3.9 ± 0.2; and 0.5 μg/kg, 5.9 ± 0.6 × $10^{-6}$ cells/ml; $r$ = 0.96; Fig. 2).

Pretreatment of rats with dexamethasone (0.5 mg/kg, s.c.) 1 h before TXB administration markedly attenuated the febrile response induced by

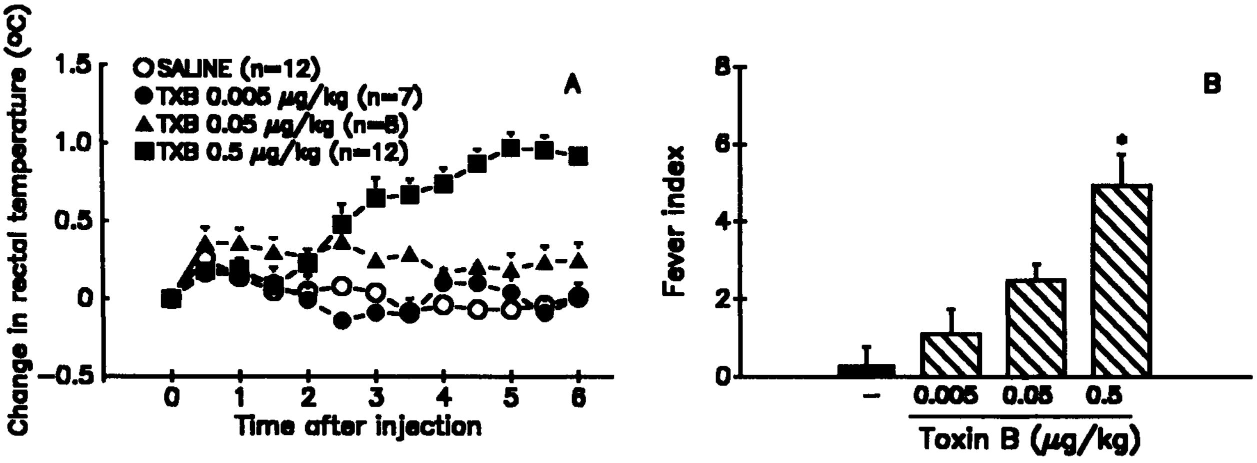

FIG. 1. Dose-dependent increase in body temperature induced by i.v. injection of different doses of *Clostridium difficile* toxin B (TXB) in rats. TXB was injected at the doses indicated and the control group received saline only. Panel A shows the time-course of the increase in body temperature induced by different doses of TXB. The values represent means ± S.E.M. of the changes in rectal temperature. Panel B shows means ± S.E.M. of fever index of the different thermal responses shown on panel A. *Significantly different from control ($p < 0.05$).

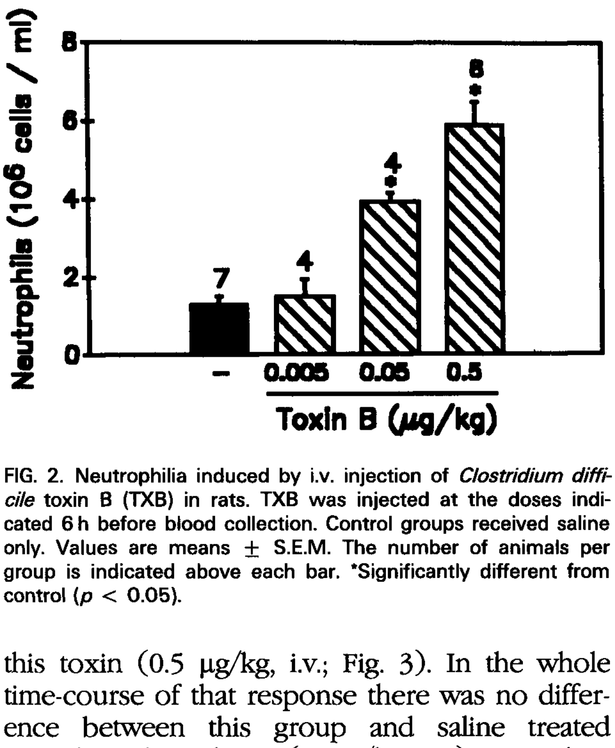

FIG. 2. Neutrophilia induced by i.v. injection of *Clostridium difficile* toxin B (TXB) in rats. TXB was injected at the doses indicated 6 h before blood collection. Control groups received saline only. Values are means ± S.E.M. The number of animals per group is indicated above each bar. *Significantly different from control ($p < 0.05$).

this toxin (0.5 μg/kg, i.v.; Fig. 3). In the whole time-course of that response there was no difference between this group and saline treated animals. Indomethacin (2 mg/kg, i.p.) injected 30 min before the TXB significantly reduced the febrile response measured between 4 and 8 h after TXB injection (Fig. 4), but did not alter the neutrophilia (control, 5.9 ± 0.6; indomethacin pretreated, 6.7 ± 0.8 × $10^{-6}$ cells/ml). The mean fever index was significantly reduced from 6.62 ± 0.90 to 2.16 ± 0.83 and from 6.46 ± 0.60 to 3.22 ± 0.46 by dexamethasone and indomethacin pretreatments, respectively.

The mortality in animals that received 0.5 μg/kg of TXB was 10.5% within 8 h, and was not modified by pretreatment with indomethacin or dexamethasone. The injection of 2.5 μg/kg of TXB (five times higher than the highest dose considered here) killed all animals within 4 h ($n = 4$).

### Discussion

This study shows that intravenous injection of TXB from *Clostridium difficile* induces dose-dependent increases in rectal temperature and neutrophilia, thus mimicking two important components of APR. To our knowledge this study represents the first demonstration of the ability of *C. difficile* toxins to induce fever.

Toxin B, at a dose of 2.5 μg/kg, induced 100% mortality in rats within 4 h of injection. A significant level of mortality induced by TXB was also found by Lyerly *et al.*,[27] where doses of 1 to 5 μg/animal of this toxin resulted in the death of about 50% of infant mice. This mortality may well be related to the ability of TXB to induce release of cytokines, such as IL-1α, IL-1β, IL-6, and TNF,[29] which are key mediators of APR[34] and septic shock.[35] Also, TXB is cytotoxic for a variety of cells, e.g. monocytes and colonic mucosal epithelial cells,[29,36] which also might contribute to the mortality caused by this toxin.

In the present study, dexamethasone markedly reduced TXB-induced fever. It is well known that dexamethasone inhibits febrile responses to a variety of pyrogenic stimuli, among them polyinosinic:polycytidilic acid, lipopolysaccharide from Gram-negative bacteria, as well as different cytokines.[4,37] This synthetic glucocorticoid has a wide range of activity and may exert its antipyretic effects by reducing the synthesis and secretion of pyrogenic cytokines and eicosanoids. It is well described that glucocorticoids suppress the cytokine or LPS-stimulated production of IL-1,[38]

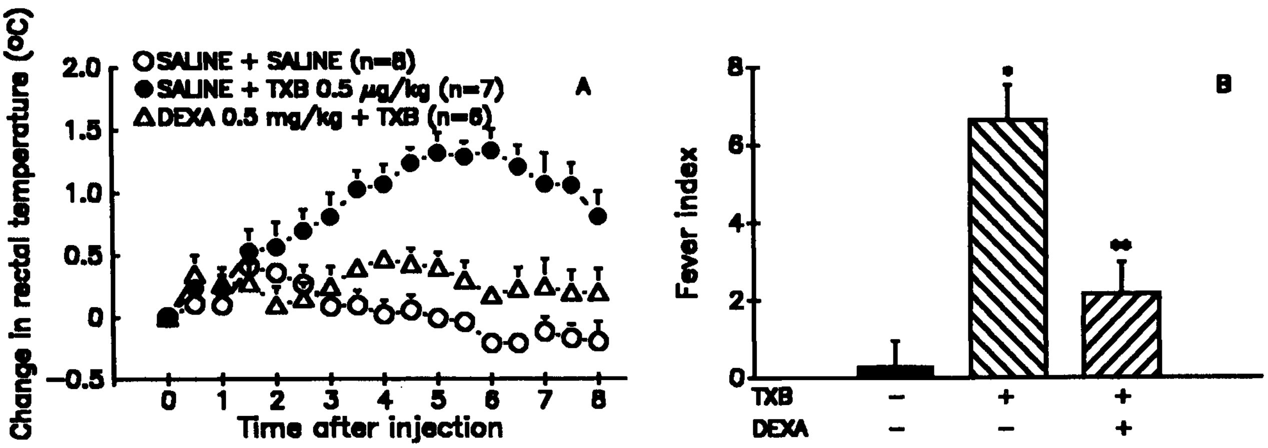

FIG. 3. Effect of dexamethasone (DEXA) pretreatment on the increase in rectal temperature induced by i.v. injection of 0.5 μg/kg of *Clostridium difficile* toxin B (TXB) in rats. DEXA or saline (control groups) was given 1 h before TXB. The different treatments are indicated in the figure. Panel A shows the time-course of the changes in body temperature for the different treatments. The values represent means ± S.E.M. of the changes in rectal temperature. Panel B shows means ± S.E.M. of fever index of the different thermal responses shown on panel A. *Significantly different from saline + saline; and **from saline + TXB, ($p < 0.05$).

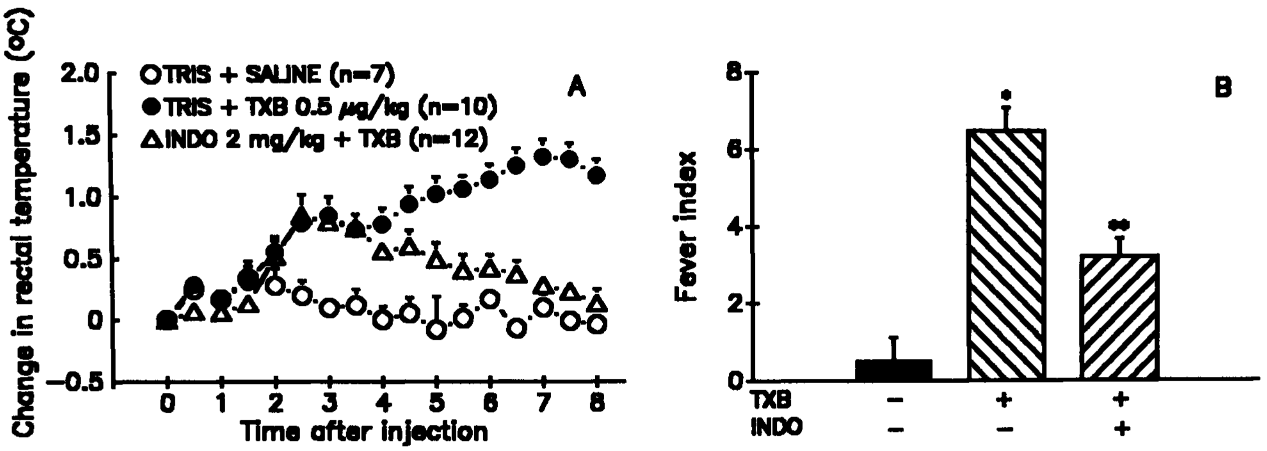

FIG. 4. Effect of indomethacin pretreatment on the increase in rectal temperature induced by i.v. injection of 0.5 μg/kg of *Clostridium difficile* toxin B (TXB) in rats. Indomethacin or Tris.HCl buffer (control groups) was given 30 min before TXB. The different treatments are indicated in the figure. Panel A shows the time-course of the changes in body temperature for the different treatments. The values represent means ± S.E.M. of the changes in rectal temperature. Panel B shows means ± S.E.M. of fever index of the different thermal responses shown on panel A. *Significantly different from Tris + saline; and ** from Tris + TXB ($p < 0.05$).

TNF,[39] IL-6 and IL-8,[40] all of which are putative mediators of the febrile response. Furthermore, the inhibition of eicosanoid synthesis by glucocorticoids results from the inhibition of expression of the inducible cyclooxygenase isoenzyme and of mobilization of membrane phospholipids via synthesis of the phospholipase $A_2$ inhibitory lipocortins.[41,42] Thus, the susceptibility of TXB-induced fever to inhibition by dexamethasone suggests that the mechanisms involved in this response might not be different from those triggered by other stimuli.

We have also demonstrated that indomethacin inhibits the febrile response, but not the neutrophilia, induced by TXB. The anti-inflammatory activities of non-steroidal anti-inflammatory drugs have been formerly ascribed to their ability to block PGs synthesis.[43] Nevertheless, besides their ability to block cyclooxygenase, new insights into their possible alternative mechanisms of action have emerged. In this regard, the antipyretic actions of indomethacin and sodium salicylate have been linked to their ability to induce release of arginine vasopressin (AVP),[44–46] which is considered an endogenous cryogen.[47] Thus, we cannot rule out either hypothesis when interpreting our results.

Since indomethacin did not change the neutrophilia induced by TXB, it is unlikely that PGs mediate this response. On the other hand Ulich *et al.*[7,8] demonstrated that the treatment of rats with indomethacin abolishes the neutrophilia induced by IL-1β, suggesting the involvement of eicosanoids in this response. Moreover, these investigators showed that stable analogues of $PGE_1$, $PGE_2$ and $PGF_{2\alpha}$ promote neutrophilia.[6] In our hands indomethacin at the same dose significantly reduced the neutrophilia induced by endotoxin from *E. coli* (unpublished data).

The true importance of each endogenous mediator to promote fever has been a point of intense debate.[1] Since PGs do not seem to mediate TXB-induced neutrophilia, it is possible that the efficacy of indomethacin to inhibit the fever induced by this toxin may also involve mechanisms unrelated to cyclooxygenase blockade, such as AVP release. Further studies are needed to help clarify this point.

In conclusion, we report that the intravenous injection of TXB from *C. difficile* induced fever in rats by a mechanism which was susceptible to inhibition by both dexamethasone and indomethacin. On the other hand, the neutrophilia induced by TXB was not inhibited by indomethacin. Therefore, TXB may be a useful tool for the understanding of the febrile response, as well as other events of APR.

### References

1. Kluger MJ. Fever: role of pyrogens and cryogens. *Physiol Rev* 1991; **71**: 93–127.
2. Minäno FJ, Sancibrian M, Vizcaino M, *et al.* Macrophage inflammatory protein-1: unique action on hypothalamous to evoke fever. *Brain Res Bull* 1990; **24**: 849–852.
3. Davatelis G, Wolpe SD, Sherry B, *et al.* Macrophage inflammatory protein-1: a prostaglandin-independent endogenous pyrogen. *Science, Wash DC* 1989; **43**: 1066–1068.
4. Zampronio AR, Souza GEP, Silva CAA, *et al.* Interleukin-8 induces fever by a prostaglandin-independent mechanism. *Am J Physiol* 1994; **266**: R1670–R1674.
5. Zampronio AR, Silva CAA, Cunha FQ, *et al.* Indomethacin blocks the febrile response induced by interleukin-8 in rabbits. *Am J Physiol* 1995; **269**: R1469–R1474.
6. Ulich TR, Dakay EB, Williams JH, Ni R-X. *In vivo* induction of neutrophilia, lymphopenia, and diminution of neutrophil adhesion by stable analogs of prostaglandins $E_1$, $E_2$ and $F_{2\alpha}$. *Am J Pathol* 1986; **124**: 53–58.
7. Ulich TR, Castillo J, Keys M, Granger G. Recombinant human alpha lymphotoxin (tumor necrosis factor-beta) induces peripheral neutrophilia and lymphopenia in the rat. *Am J Pathol* 1987; **128**: 5–12.
8. Ulich TR, Castillo J, Keys M, *et al.* Kinetics and mechanisms of recombinant human interleukin 1 and tumor necrosis factor-α-induced changes in circulating numbers of neutrophil and lymphocytes. *J Immunol* 1987; **139**: 3406–3415.
9. Ulich TR, Castillo J, Guo K. *In vivo* hematologic effects of recombinant interleukin-6 on hematopoesis and circulating numbers of RBCs and WBCs. *Blood* 1989; **73**: 108–111.
10. Kajita T, Hugli TE. C5a-induced neutrophilia. *Am J Pathol* 1990; **137**: 467–477.
11. Hechtman DH, Cybulsky MI, Fuchs HJ, *et al.* Intravascular IL-8. *J Immunol* 1991; **147**: 883–892.
12. Bartlett JG. *Clostridium difficile*: pseudomembranous colitis and antibiotic-associates diarrhea. In: Gorbach SL, ed. *Infectious Diarrhea.* Boston: Blackwell, 1986; 157.
13. Lyerly DM, Krivan HC, Wilkins TD. *Clostridium difficile*: its disease and toxins. *Clin Microbiol Rev* 1988; **1**: 1–18.
14. Kelly CP, Pothoulakis C, LaMont JT. *Clostridium difficile* colitis. *New Engl J Med* 1994; **330**: 257–262.
15. Triadafilopoulos G, Hallstone AE. Acute abdomen as the first presentation of pseudomembranous colitis. *Gastroenterology* 1991; **101**: 685–691.
16. Andrejak M, Schmit JL, Tondriaux A. The clinical significance of antibiotic-associated pseudomembranous colitis in the 1990s. *Drug Saf* 1991; **6**: 339–349.
17. Pieri L, Mazzeo S, Caramella D, *et al.* La diagnostica per immagini nella colite pseudomembranosa. [Imaging diagnosis in pseudomembranous colitis]. *Radiol Med Torino* 1994; **87**: 775–782.
18. Osebold WR, Cohen AN, Gillum MD, *et al.* Postoperative *Clostridium difficile* pseudomembranous colitis in idiopathic scoliosis. A brief clinical report. *Spine* 1993; **18**: 2549–2552.
19. Clarke HJ, Jinnah RH, Byank RP, Cox QC. *Clostridium difficile* infection in orthopaedic patients. *J Bone Joint Surg Am* 1990; **72**: 1056–1059.
20. Viscidi R, Laughon BE, Yolken R, *et al.* Serum antibody response to toxins A and B of *Clostridium difficile. J Infect Dis* 1983; **148**: 93–100.
21. Lyerly DM, Roberts MD, Phelps CD, Wilkins TD. Purification and properties of toxin A and B of *Clostridium difficile. FEMS Microbiol Lett* 1986; **33**: 31–35.
22. Sauerborn M, von Eichel-Streiber C. Nucleotide sequence of *Clostridium difficile* toxin A. *Nucleic Acids Res* 1990; **18**: 1629.
23. Dove CH, Wang S-Z, Price SB, *et al.* Molecular characterization of the *Clostridium difficile* toxin A gene. *Infect Immun* 1990; **58**: 480–488.
24. Barroso LA, Wang S-Z, Phelps CJ, *et al.* Nucleotide sequence of *Clostridium difficile* toxin B gene. *Nucleic Acids Res* 1990; **18**: 4004.
25. Lima AAM, Lyerly DM, Wilkins TD, *et al.* Effects of *Clostridium difficile* toxins A and B in rabbit small and large intestine *in vivo* and on cultured cells *in vitro. Infect Immun* 1988; **56**: 582–588.
26. Banno Y, Kobayashi T, Kono H, *et al.* Biochemical characterization and biologic actions of two toxins (D-1 and D-2) from *Clostridium difficile. Rev Infect Dis* 1984; **6**: 511–520.
27. Lyerly DM, Lockwood DE, Richardson SH, Wilkins TD. Biological activities of toxins A and B of *Clostridium difficile. Infect Immun* 1982; **35**: 1147–1150.
28. Lyerly DM, Saum KE, MacDonald DK, Wilkins TD. Effects of *Clostridium difficile* toxins given intragastrically to animals. *Infect Immun* 1985; **47**: 349–352.
29. Flegel WA, Müller F, Däubener W, *et al.* Cytokines response by human monocytes to *Clostridium difficile* toxin A and toxin B. *Infect Immun* 1991; **59**: 3659–3666.
30. Miller PD, Pothoulakis C, Baeker TR, *et al.* Macrophage-dependent stimulation of T cell-depleted spleen cells by *Clostridium difficile* toxin A and calcium ionophore. *Cell Immunol* 1990; **126**: 155–163.
31. Guerrant RL. Lessons from diarrheal diseases: demography to molecular pharmacology. *J Infect Dis* 1994; **169**: 1206–1218.
32. Gordon CJ. Thermal biology of the laboratory rat. *Physiol & Behav* 1990; **47**: 963–991.
33. Sullivan NM, Pellet S, Wilkins TD. Purification and characterization of toxins A and B of *Clostridium difficile. Infect Immun* 1982; **35**: 1032–1040.
34. Kushner I. The acute phase response: an overview. *Meth Enzymol* 1988; **163**: 373–383.
35. Tracey KJ, Cerami A. Tumor necrosis factor: a pleiotropic cytokine and therapeutic target. *Annu Rev Med* 1994; **45**: 491–503.
36. Rieger M, Sedivy R, Pothoulakis C, *et al. Clostridium difficile* toxin B is more potent than toxin A in damaging human colonic epithelium *in vitro. J Clin Invest* 1995; **95**: 2004–2011.
37. Abul H, Davidson J, Milton AS, Rotondo D. Dexamethasone pretreatment is antipyretic toward polyinosinic:polycytidilic acid, lipopolysaccharide and interleukin 1/endogenous pyrogen. *Naunyn-Schmiedberg's Arch Pharmacol* 1987; **335**: 305–309.
38. Staruch MJ, Wood D. Reduction of serum interleukin-1-like activity after treatment with dexamethasone. *J Leukocyte Biol* 1985; **37**: 193–207.
39. Waage A, Bakke O. Glucocorticoids suppress the production of tumor necrosis factor by lipopolysaccharide-stimulated human monocytes. *Immunology* 1988; **63**: 299–302.
40. Tobler A, Meier R, Seitz M, *et al.* Glucocorticoids down-regulate gene expression of GM-CSF, NAP-1/IL-8, and IL-6, but not of M-CSF in human fibroblasts. *Blood* 1992; **79**: 45–51.
41. Goppelt-Struebe M, Wolter D, Resch K. Glucocorticoids inhibit prostaglandin synthesis not only at the level of phospholipase $A_2$ but also at the level of cyclo-oxygenase/PGE isomerase. *Br J Pharmacol* 1989; **98**: 1287–1295.
42. Flower RJ, Rothwell NJ. Lipocortin-1: cellular mechanisms and clinical relevance. *TiPS* 1994; **15**: 71–76.
43. Vane JR. Inhibition of prostaglandin synthesis as a mechanism of action for aspirin-like drugs. *Nature New Biol* 1971; **231**: 272–273.
44. Wilkinson MF, Kasting NW. Central vasopressin V1-receptors mediate indomethacin-induced antipyresis in rat. *Am J Physiol* 1989; **256**: R1164–R1168.
45. Wilkinson MF, Kasting NW. Central vasopressin V1-blockade prevents salicylate but not acetaminophen antipyresis. *J Appl Physiol* 1990; **68**: 1793–1798.
46. Wilkinson MF, Kasting NW. Vasopressin release within the ventral septal area of the rat brain during drug-induced antipyresis. *Am J Physiol* 1993; **264**: R1133–R1138.
47. Pittman QJ, Poulin P, Wilkinson MF. Role of neurohypophysial hormones in temperature regulation. *Annals NY Acad Sci* 1993; **689**: 375–381.

ACKNOWLEDGEMENTS. We gratefully acknowledge Dr G. A. Rae (Federal University of Santa Catarina) for helpful discussions and review of the manuscript. This work was supported by FAPESP and CNPq. R.A.C has a fellowship from CAPES (Brazil).

**Received 29 December 1995; accepted in revised form 22 February 1996**
